# Genetic selection for growth, wood quality and resin traits of potential Slash pine for multiple industrial uses

**DOI:** 10.48130/forres-0024-0020

**Published:** 2024-06-20

**Authors:** Xianyin Ding, Yini Zhang, Jiaming Sun, Zifeng Tan, Qinyun Huang, Shu Diao, Yadi Wu, Qifu Luan, Jingmin Jiang

**Affiliations:** 1 Research Institute of Subtropical Forestry, Chinese Academy of Forestry, Hangzhou 311400, China; 2 National Forestry and Grassland Engineering Technology Research Center of Exotic Pine Cultivation, Hangzhou 311400, China

**Keywords:** Slash pine, Industrial uses, Non-destructive assessment, Genetic variation, Genetic gain, Multi-trait combined selection

## Abstract

This study aims to understand the genetic basis of key industrial traits in Slash pine (*Pinus elliottii* Engelm. var. *elliottii*) to enhance improvement efficiency. Detailed analyses were conducted on inter-family differences, genetic parameters, correlations, and breeding values (BVs) for growth, wood properties, and resin traits of Slash pine planted in Changle Forest Farm of Hangzhou, leading to the identification of elite families. It indicates that growth traits are primarily influenced by environmental effects, while wood properties exhibit a significant impact of genetic effects. The variation in resin traits arises from both genetic and environmental effects. Notably, Beta-pinene exhibits the highest variability and genetic gains among the traits analyzed. The family heritability ranges for growth, wood properties, and resin traits are 0.543−0.794, 0.870−0.885, and 0.285−0.695, respectively. Significant positive correlations are evident between growth and resin traits, while a negative correlation is observed between growth and wood properties. Elite families identified through single-trait and multi-trait combined selection are 8−126 for growth traits, 2−325 and 0−373 for wood properties, and 8−131 for resin traits. The average genetic gains for these elite families are 7.44%, 7.17%, and 8.84%, respectively. These findings provide valuable insights for high-generation breeding of Slash pine and lay a genetic foundation for formulating effective breeding strategies for conifers.

## Introduction

Wood, known for its renewable and eco-friendly properties, serves diverse purposes in construction, furniture, and pulp production^[1]^. Key growth parameters like diameter at breast height (DBH) and tree height (Ht), alongside wood properties such as wood density (WD) and modulus of elasticity (MOE), are essential for breeding programs targeting industrial production^[2,3]^. Traditional assessment methods for these traits are costly, time-intensive, and destructive, posing challenges in forestry research^[4]^. However, non-destructive wood quality assessment technologies like Pilodyn and Resistograph offer a promising solution, enabling rapid and accurate target detection in a cost-effective and high-throughput manner^[5,6]^. Resin, a viscous fluid mixture of secondary metabolites comprises turpentine rich in monoterpenes and sesquiterpenes, and rosin predominantly composed of diterpenes^[7]^. Turpentine finds applications in fragrance, pharmaceuticals, and food additives, while rosin is extensively used in agrochemicals and adhesives^[8,9]^. Resin derivatives have diversified applications in biodegradable batteries, green plastics, and petroleum alternatives^[9,10]^. Each resin component serves distinct purposes in industrial applications^[11,12]^. For example, pinene, the predominant turpentine, is utilized in natural insecticides, pharmaceuticals, and food additives^[13−16]^. Among pinene isomers, Alpha-pinene is the most abundant, while Beta-pinene holds significant value in the chemical industry^[17]^. Slash pine (*Pinus elliottii* Engelm. var. *elliottii*) resin, characterized by its high Beta-pinene content is of considerable industrial value^[18,19]^.

Genetic variation is crucial for genetic improvement, providing insights into trait breeding potential^[12]^. In forestry genetic improvement, breeders often aim to enhance multiple traits simultaneously. Therefore, investigating the relationship between growth traits and wood properties is crucial. Studies have indicated a positive genetic correlation between growth traits and wood properties in *P. taeda*^[20]^ and *P. massoniana*^[21]^. However, the correlation between growth traits and WD in *P. contorta*^[22]^ is not significant, contrasting with other research suggesting a negative correlation between growth trait and wood properties in *P. taeda*^[23]^, *Larix kaempferi*^[24]^, and *P. radiata*^[25]^. Consequently, achieving rapid wood maturation through genetic improvement for growth traits may potentially have adverse effects on wood quality^[26,27]^. Additionally, research on resin traits has revealed significant negative correlations between Alpha- and Beta-pinene. Furthermore, resin yield and resin basic density exhibit a highly significant positive correlation, while turpentine content shows a highly significant negative correlation with resin basic density^[28]^. Understanding these connections is crucial for implementing multi-trait combined selection, optimizing breeding strategies, and achieving balanced improvements in growth-wood-resin traits^[18]^.

However, economically valuable traits in tree breeding programs typically exhibit polygenic variation and are strongly influenced by environmental factors. Almost all commercially important tree species have reported significant Genotype-by-Environment interactions (G × E), including Scots pine^[29]^, Eucalyptus^[30]^, Douglas fir^[31]^, Norway spruce^[32]^, and Poplar^[33]^. Multi-trait combined selection has the potential to mitigate the impact of the environment on genotype stability and expedite the breeding process^[34]^. Nonetheless, addressing linkage disequilibrium (LD) while simultaneously selecting for multiple desirable traits has led to various methods for multi-trait combined selection^[35]^, such as index selection^[36]^, sequential selection^[37]^, and multi-factor comprehensive evaluation^[38]^. Principal component analysis (PCA) is one such method, revealing the intrinsic characteristics of test materials by extracting factors from the multifactorial relationships between components and performance, which has demonstrated efficacy in *Larix kaempferi*^[39]^ and *Camellia chekiangoleosa*^[40]^.

Slash pine plays a crucial role in providing wood and resin material, with genetic improvement efforts primarily targeting growth rates, wood quality, and oleresin yield (OY)^[41]^. This study aims to: (1) investigate the varied G × E effects on growth, wood properties, and resin traits; (2) estimate the heritability, correlations, and breeding values of these traits; and (3) employ multi-trait combined selection to identify elite families. These findings may enhance the efficiency of genetic improvement for key industrial traits in Slash pine and accelerate its high-generation breeding process.

## Materials and methods

### Plant materials and experimental design

The Slash pine clonal seed orchard (located at 30°20' N, 119°50' E) comprises 300 elite clone genotypes selected from Slash pine provenance test forests in seven provinces: Guangdong, Fujian, Jiangxi, Jiangsu, Hunan, Hubei, and Zhejiang. These clones, all exhibiting elite growth performance and unrelated to each other, were propagated in April 1976 by grafting robust branches from the crown of selected elite trees onto 2-year-old Slash pine rootstocks using the method of cambial layer grafting.

In 1992, seeds from the clonal families of the orchard were collected, and seedlings were raised in 1993. In 1994, a clonal stand test forest was established at coordinates 30°27' N, 119°49' E. The test forest comprises 33 families, arranged in a completely randomized design with six offspring in single-row plots and six replications (blocks), with trees spaced 2 m apart within rows and rows spaced 3 m apart. The local annual average precipitation is 1,480 mm, and the annual average temperature is 17.0 °C. The planting site is characterized by gently sloping hills with yellow-red soil of moderate fertility. Thinning was carried out in the test forest after 10 years of establishment. The test forest had never been tapped for resin before the commencement of this experiment.

In July 2019, phenotypic surveys were conducted on four blocks of the test forest. The three trees with the largest DBH were measured in each plot, and trees with obvious damage were excluded. This resulted in a total of 240 individual phenotype data collected (Supplemental Table S1).

### Growth traits phenotyping

In this study, the phenotypic traits and survey methods for the growth traits of Slash pine are as follows:

(1) DBH: Measured with a caliper at a height of 1.3 m from the ground on the trunk.

(2) Ht and height under the crown (Huc): Measured using a height measuring pole.

(3) Crown width: Diameter of the crown measured in both east-west and north-south directions, with the average result taken and the area calculated.

(4) Average annual ring width (ARW): ARW was measured using the Resistograph-IML-RESIPD500 (IML-RESI GmbH, Taufkirchen, Germany). The drill was inserted vertically at the tree's DBH, recording depth and amplitude for each 0.1 mm. Peaks and valleys correspond to annual growth cycles. Data selection excluded bark-induced irregularities, focusing on distinct resistance peaks. The ARW and resistance value (Mountain peak, MP) were calculated. Due to needle height, the first-year ring is often unclear, starting ring counting from the outermost. Each tree's data covered 24 years for RW and yearly MP. ARW and AMP were recorded, followed by data analysis using PDToolsPro software. The detailed operational method has been previously described in our earlier research^[42]^.

### Wood properties phenotyping

(1) WD: Using a growth increment borer with a diameter of 5 mm, cores were extracted from the trees at breast height in the north-south direction, ensuring that the borehole penetrated through the pith of the tree. Basic density ($ \rho $) was determined using the saturated moisture content method^[43]^:

1 $ \rho =\dfrac{1}{\dfrac{{\mathrm{M}}_{\mathrm{w}}}{{\mathrm{M}}_{\mathrm{d}}}-0.3464} $

In Eqn (1), $ \rho $ is the basic density of wood (g·cm^−3^), $ {\mathrm{M}}_{\mathrm{w}} $ is the mass of the wood core when saturated with water, $ {\mathrm{M}}_{\mathrm{d}} $ is the mass of wood core when completely dry.

(2) Determination of stress wave velocity $ v $ and *MOE*: The Hitman ST300 (Fibre-gen, Christchurch, New Zealand) was utilized to measure the $ v $ of the samples. The specific method is detailed in the research findings of Zhang et al.^[44]^. Subsequently, the *MOE* of the samples was calculated using Eqn (2) based on the $ \rho $ and $ v $.

2 $ MOE=\rho {v}^{2} $

(3) Pilodyn (Pd) detection: Using Pilodyn (6J, PROCEQ, Switzerland), cores were extracted from the lower 2 cm of the stem of individual trees in both the south and north directions. Two Pd resistance values (Ps and Pn) were measured, and only values with a difference of no more than 2 mm were considered valid. Otherwise, the two values with the smallest difference were selected. The average resistance values in the south and north directions were denoted as Pd.

(4) The AMP data acquisition method is as described in the section 'Growth traits phenotyping'.

### Resin traits phenotyping

(1) OY: A specialized plastic tube with a diameter of 1.8 mm and a volume of 15 mL is fixed in the borehole on the sunny side of the trunk. After waiting for 24 h, the resin is collected, and then its yield is measured using a balance. The detailed installation method is described in Li et al.^[45]^.

(2) Relative amount of resin components: Gas chromatography experiments were carried out with a GC 6890 gas chromatograph (Agilent 5975B, Santa Clara, CA, USA) equipped with a DB-5MS capillary column cross-linked with 5% Ph Me silicone (60 m/0.25 mm/0.25 mm) and coupled with a Hewlett Packard GC 5975B mass spectrometer. The qualitative and quantitative analysis of resin composition with the chromatographic conditions was as follows: GC: 0.05 g of pine resin was dissolved in 0.5 mL of ethyl alcohol containing 50 μL tetramethylammonium hydroxide. The initial column temperature was 60 °C, held for 2 min, increased at 8 °C·min^−1^ to 80 °C, and reached a maximum of 280 °C at a rate of 2 °C·min^−1^ for 5 min. The helium gas flow was set at 1 mL·min^−1^. The temperature of the injector was 260 °C, and the volume was 1 μL with a 1/50 split ratio. Mass spectra were recorded under electron impact ionization at an electron energy of 70 eV in the range from m/z 30 to 600 along with solvent delay for 3 min.

Resin compositions were identified by matching experimental fragmentation patterns in mass spectra with the NIST08 database through the data processing system of Agilent Chem Station and then compared with the relevant literature. Monoterpene content was determined by isobutylbenzene content, and diterpene content was determined by heptadecanoic acid content. The resin component contents were calculated by comparing their peak areas. We then selected four components believed to have broad industrial utility for subsequent analysis, including two monoterpenes (Alpha- and Beta-pinene) and two diterpenes (Abietic and Levoprimaric acid).

### Statistical analysis method

The linear model for each observation of the resin component trait *y*_*ijk*_ in each tree was shown as follows:

3 $ \mathit{y} _{ \mathit{ijk} } = \mu+{f} _{ \mathit{i} } \mathit{+b} _{ \mathit{j} } \mathit{+fb} _{ \mathit{ij} } \mathit{+e} _{ \mathit{ijk} } $

Where the observed values (*y*_*ijk*_) for individual plants within each family (*i*), block (*j*), and specific plant (*k*) are considered, The *μ* represents the average value across all observations. Family effects are denoted by *f*_*i*_. Similarly, *b*_*j*_ refers to block effects. The interaction effect between family and block is represented by *fb*_*ij*_. Environmental error effects (*e*_*ijk*_) encapsulate random variations and unforeseen factors affecting individual plant observations.

The genetic variation analysis employed the Restricted Maximum Likelihood (REML) method to fit the Generalized Linear Mixed Model (GLMM). Cuevas et al. provided a detailed description of this approach, wherein the model equations are derived by stacking the vectors for all individual plants^[46]^:

4 $ \mathrm{y}=\mathrm{X}\mathrm{m}+{\mathrm{Z}}_{1}\mathrm{f}+{\mathrm{Z}}_{2}\mathrm{b}+{\mathrm{Z}}_{3}\mathrm{f}\mathrm{b}+\mathrm{e} $

Where the vector y represents the observed values of the overall phenotype. The vector m denotes the population mean values, while b, f, and e represent the vectors of block effects, family effects, family and block interaction effects, and random error effects, respectively. The design matrices X, Z_1_, Z_2_ and Z_3_ are corresponding correlation matrices used to link the observed values with their respective effects. We define the expected value vector (E) and the discrete matrix (Var) as:

5 $ E\left[\mathrm{y}\right]=\mathrm{ }\mathrm{X}\mathrm{m} $

6 $ Var\left[\mathrm{f}\right]={\mathrm{Z}}_{1}\otimes {\mathrm{F}}_{0} $

7 $ Var\left[\mathrm{b}\right]={\mathrm{Z}}_{2}\otimes {\mathrm{B}}_{0} $

8 $ {Var}\left[\mathrm{f}\mathrm{b}\right]={{\mathrm{Z}}_{1}\mathrm{Z}}_{2}\otimes {{\mathrm{F}}_{0}\mathrm{B}}_{0} $

9 $ Var\left[\mathrm{e}\right]=\mathrm{Z}\oplus{\mathrm{R}}_{0} $

and

10 $ {\mathrm{F}}_{0}=\left[\begin{array}{cc}{\sigma }_{{f}_{1}}^{2}& {\sigma }_{{f}_{1}{f}_{2}}\\ {\sigma }_{{f}_{2}{f}_{1}}& {\sigma }_{{f}_{2}}^{2}\end{array}\right] $

11 $ {\mathrm{B}}_{0}=\left[\begin{array}{cc}{\sigma }_{{b}_{1}}^{2}& {\sigma }_{{b}_{1}{b}_{2}}\\ {\sigma }_{{b}_{2}{b}_{1}}& {\sigma }_{{b}_{2}}^{2}\end{array}\right] $

12 $ {{\mathrm{F}}_{0}\mathrm{B}}_{0}=\left[\begin{array}{cc}{\sigma }_{{f}_{1}{b}_{2}}^{2}& {\sigma }_{{f}_{1}{b}_{2}}\\ {\sigma }_{{f}_{2}{b}_{1}}& {\sigma }_{{f}_{2}{b}_{2}}^{2}\end{array}\right] $

13 $ {\mathrm{R}}_{0}=\left[\begin{array}{cc}{\sigma }_{{e}_{1}}^{2}& {\sigma }_{{e}_{1}{e}_{2}}\\ {\sigma }_{{e}_{2}{e}_{1}}& {\sigma }_{{e}_{2}}^{2}\end{array}\right] $

Where the $ \otimes $ and $ \oplus $ are vector product and vector addition respectively; $ {\sigma }_{{f}_{i}}^{2} $, $ {\sigma }_{{b}_{i}}^{2} $, $ {\sigma }_{{f}_{i}{b}_{i}}^{2} $ and $ {\sigma }_{{e}_{i}}^{2} $ represent the variances of the block effect, family effect, family × block interaction effect, and environmental error effect for trait *i*, respectively; $ {\sigma }_{{b}_{i}{b}_{j}} $, $ {\sigma }_{{f}_{i}{f}_{j}} $ and $ {\sigma }_{{e}_{i}{e}_{j}} $ are the covariances effects of block, family and environmental errors for traits *i* and *j*. The variance component of the model was used to calculate the family mean heritability (*H*^*2*^) and individual narrow-sense heritability (*h*^*2*^):

14 $ {H}_{i}^{2}=\dfrac{{\sigma }_{{f}_{i}}^{2}+{\sigma }_{{b}_{i}}^{2}}{{\sigma }_{{f}_{i}}^{2}+{\sigma }_{{b}_{i}}^{2}+{\sigma }_{{f}_{i}{b}_{i}}^{2}/{n}_{b}+{\sigma }_{{e}_{i}}^{2}/{n}_{b}{n}_{k}} $

15 $ {h}_{i}^{2}=\dfrac{4({\sigma }_{{f}_{i}}^{2}+{\sigma }_{{b}_{i}}^{2})}{{\sigma }_{{f}_{i}}^{2}+{\sigma }_{{b}_{i}}^{2}+{\sigma }_{{f}_{i}{b}_{i}}^{2}+{\sigma }_{{e}_{i}}^{2}} $

Phenotypic correlation $ {r}_{{p}_{ij}} $ and genetic correlation $ {r}_{{g}_{ij}} $ of traits *i* and *j*:

16 $ {r}_{{p}_{ij}}=\dfrac{{\sigma }_{{f}_{ij}}+{{\sigma }_{{b}_{ij}}}+\sigma _{{e}_{ij}}}{\sqrt{\left({\sigma }_{{f}_{i}}^{2}+{{\sigma }_{{b}_{i}}^{2}+{\sigma }_{{f}_{i}{b}_{i}}^{2}}+\sigma _{{e}_{i}}^{2}\right)\left({\sigma }_{{f}_{j}}^{2}+{\sigma }_{{b}_{j}}^{2}+{\sigma }_{{f}_{j}{b}_{j}}^{2}+{\sigma }_{{e}_{j}}^{2}\right)}} $

17 $ {r}_{{g}_{ij}}=\dfrac{{\sigma }_{{f}_{ij}}{+\sigma }_{{b}_{ij}}}{\sqrt{\left({\sigma }_{{f}_{i}}^{2}+{\sigma }_{{b}_{i}}^{2}\right)\left({\sigma }_{{f}_{j}}^{2}+{\sigma }_{{b}_{j}}^{2}\right)}} $

Among them, $ {\sigma }_{{f}_{ij}} $, $ {\sigma }_{{b}_{ij}} $, $ {\sigma }_{{e}_{ij}} $ represent the covariance of family effect, block effect and environmental errors effect for traits *i* and *j*; $ {\sigma }_{{f}_{i}}^{2} $ and $ {\sigma }_{{f}_{j}}^{2} $ represent the family variance estimate of traits *i* and *j*; $ {\sigma }_{{b}_{i}}^{2} $ and $ {\sigma }_{{b}_{j}}^{2} $ represent the block variance estimate of traits *i* and *j*; $ {\sigma }_{{e}_{i}}^{2} $ and $ {\sigma }_{{e}_{j}}^{2} $ represent the environmental errors variance estimate of traits *i* and *j*. $ {\sigma }_{{b}_{i}{f}_{i}}^{2} $ and $ {\sigma }_{{b}_{j}{f}_{j}}^{2} $ represent the variances of family × block interaction effect; The $ {n}_{b} $ and $ {n}_{k} $ are block numbers and tree numbers per family, respectively.

Coefficient of variation (CV/%):

18 $ {\mathrm{CV}}=100\overline{x}/\sigma $

Coefficient of phenotypic variation (*I*_*p*_) and Coefficient of genetic Variation (*I*_*g*_) :

19 $ {I}_{p}=\sqrt{{\sigma }_{p}^{2}}\Big/\overline{x} $

20 $ {I}_{g}=\sqrt{{\sigma }_{g}^{2}}\Big/\overline{x} $

Among them, $ \sqrt{{\sigma }_{p}^{2}} $ is the square root of the phenotypic variance component; $ \sqrt{{\sigma }_{g}^{2}} $ is the square root of the genetic variance component; $ \overline{x} $ is the trait mean value.

The genetic gain ($ \Delta G $) is estimated by equation (17) :

21 $ \Delta G=i\cdot H\cdot {\sigma }_{g}/t $

Among them, *i* is the selection intensity; $ H $ is represented by the square root of the heritability; $ {\sigma }_{g} $ represents the square root of the additive genetic variance; *t* represents the breeding period. In this study, we employed varying selection intensities, specifically 1.40, 1.75, and 2.06, to simulate diverse selective pressures.

Breeding value (BV) is estimated by the best linear unbiased prediction (BLUP) model.

PCA was employed in the process of conducting multi-trait combined selection of elite families. The principal components (PCs) for growth traits, wood properties, and resin traits were derived from the breeding values associated with each trait. Subsequently, we computed the loadings of each trait across various PCs, along with eigenvalues, eigenvectors, contribution rates, and cumulative contribution rates for each PC. Elite families were selected based on the comprehensive scores obtained from the PC factors (*F*_*total*_) of each family.

### Statistical software

All data analyses were based on R software^[47]^. The analysis of variance was calculated by the function aov, the pedigree relationship matrix was calculated by the R language 'pedigreemm' software package^[48]^, and the genetic parameters and breeding values were estimated using the R language 'sommer' software package^[49]^. PCA analysis was performed by the function prcomp and the software package 'psych'^[50]^. All result visualizations were implemented using the R language 'ggplot2' software package^[51]^.

## Results

### Descriptive statistics and variance analysis of various traits

Table 1 presents statistical results for growth, wood properties, and resin traits in the progeny test stand of Slash pine half-sib families. Growth traits, including DBH, Ht, Huc, Crown, and ARW, had average values of 19.766 cm, 16.773 m, 9.439 m, 3.469 m^2^, and 4.254 mm, respectively, with CV ranging from 15.724% to 26.931%. For wood properties, Pd, MOE, and AMP had average values of 19.836 mm, 3.006 GPa, and 0.415%, with CVs ranging from 10.335% to 29.488%. Regarding resin traits, average values for OY, Alpha-pinene, Beta-pinene, Abietic acid, and Levopimaric acid were 1.838 g, 14.790 μg·g^−1^, 10.661 μg·g^−1^, 8.183 μg·g^−1^, and 21.631 μg·g^−1^, respectively. Beta-pinene exhibited the highest CV at 42.126%, while levopimaric acid had the lowest at 14.868%. Significant differences (*p* < 0.05) were observed in all traits among families. Moreover, except for DBH and ARW, all other traits were significantly influenced by the block effect. Additionally, Ht, Crown, AMP, Alpha-pinene, and Beta-pinene exhibited effects from family, block, and their interaction.

**Table 1:** Results of descriptive statistics and variance analysis.

Traits	Mean	SD	CV/%	F-statistic		
				Family	Block	Block × family
DBH (cm)	19.766	4.026	20.370	1.518*	0.616	0.894
Ht (m)	16.773	2.637	15.724	2.901**	7.738**	1.902*
Huc (m)	9.439	2.334	24.730	1.393*	5.102**	0.875
Crown (m^2^)	3.469	0.934	26.931	1.529*	3.362*	1.463*
ARW (mm)	4.254	0.818	19.228	1.477*	0.427	0.622
Pd (mm)	19.836	2.050	10.335	2.637**	4.351**	1.007
MOE (Gpa)	3.006	0.368	12.244	1.750*	3.842*	1.239
AMP (%)	0.415	0.122	29.488	2.731**	2.902**	1.446*
OY (g)	1.838	0.710	38.622	1.618*	1.213*	0.972
Alpha_pinene (μg·g^−1^)	14.790	4.301	29.078	1.184*	0.649*	0.771*
Beta_pinene (μg·g^−1^)	10.661	4.491	42.126	1.274*	1.241*	0.929*
Abietic_acid (μg·g^−1^)	8.183	2.994	36.596	0.887*	1.221*	1.094
Levopimaric_acid (μg·g^−1^)	21.631	3.216	14.868	1.341*	2.703*	1.212
** denotes the significance level of *p* < 0.01, while * indicates *p* < 0.05.						

**Figure 1:**
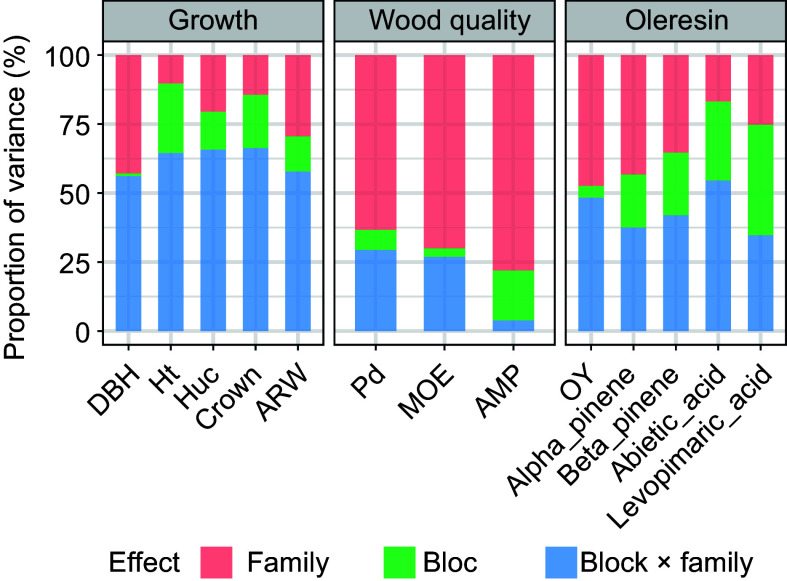
The proportion of variance components of different effects for each trait.

The variation in growth traits primarily stems from the interaction between family and block effects (Fig. 1). Notably, the largest effect influenced by environmental factors is the Crown variance at 66.28%, indicating a predominant influence of the environment on Slash pine growth traits. Conversely, variation in wood properties is primarily attributed to family effects, with a proportion of 78.08% for AMP, suggesting a lesser impact of environmental factors. For resin traits, environmental effects emerge as the primary source of variation, while the OY trait shows nearly equal contributions from genetic and environmental factors at 47.40% and 48.33%, respectively.

### Estimation of genetic parameters of various traits

Based on the variance components, genetic parameters for various traits were estimated (Table 2). Among the three types of traits, Crown, AMP, and Beta-pinene exhibited relatively greater phenotypic and genetic variability, while Ht, Pd, and Levopimaric acid showed lower variability. Ht, Pd, and Beta-pinene demonstrated relatively high family heritabilities of 0.794, 0.855, and 0.695, respectively, indicating significant genetic control. Notably, all three wood properties exhibited relatively high genetic control. The individual heritability of all traits aligned with the family heritability. Particularly, Ht, Pd, and Beta-pinene achieved the highest genetic gain, reaching 11.4%, 11.1%, and 14.8%, respectively, at a 5% selection rate. These findings underscore the exceptional breeding potential of Ht, Pd, and Beta-pinene among all traits in this study.

**Table 2:** Genetic parameter estimation results for various traits.

Traits	$ {I}_{p} $	$ {I}_{g} $	$ {h}_{i}^{2} $	$ {H}_{i}^{2} $	$ \Delta G $		
					*r* = 0.05 *i* = 2.06	*r* = 0.10 *i* = 1.75	*r* = 0.20 *i* = 1.40
DBH	0.205	0.041	0.172 (0.017)	0.593 (0.296)	0.107	0.091	0.073
Ht	0.155	0.092	0.355 (0.021)	0.794 (0.151)	0.114	0.097	0.077
Huc	0.240	0.091	0.145 (0.017)	0.543 (0.033)	0.052	0.045	0.036
Crown	0.268	0.115	0.183 (0.017)	0.610 (0.284)	0.026	0.022	0.017
ARW	0.194	0.113	0.338 (0.024)	0.781 (0.181)	0.035	0.030	0.024
Pd	0.099	0.072	0.524 (0.024)	0.885 (0.100)	0.111	0.094	0.075
MOE	0.121	0.063	0.273 (0.019)	0.870 (0.219)	0.015	0.012	0.010
AMP	0.297	0.170	0.281 (0.020)	0.874 (0.214)	0.005	0.005	0.004
OY	0.379	0.150	0.157 (0.023)	0.566 (0.042)	0.017	0.015	0.012
Alpha_pinene	0.290	0.143	0.244 (0.020)	0.694 (0.234)	0.146	0.124	0.099
Beta_pinene	0.421	0.202	0.245 (0.020)	0.695 (0.251)	0.148	0.126	0.101
Abietic_acid	0.366	0.061	0.173 (0.013)	0.285 (0.049)	0.022	0.019	0.015
Levopimaric_acid	0.147	0.061	0.172 (0.010)	0.592 (0.031)	0.084	0.071	0.057
*r* represents the selection rate, and *i* represents the selection intensity.							

### Correlation analysis of various traits

**Figure 2:**
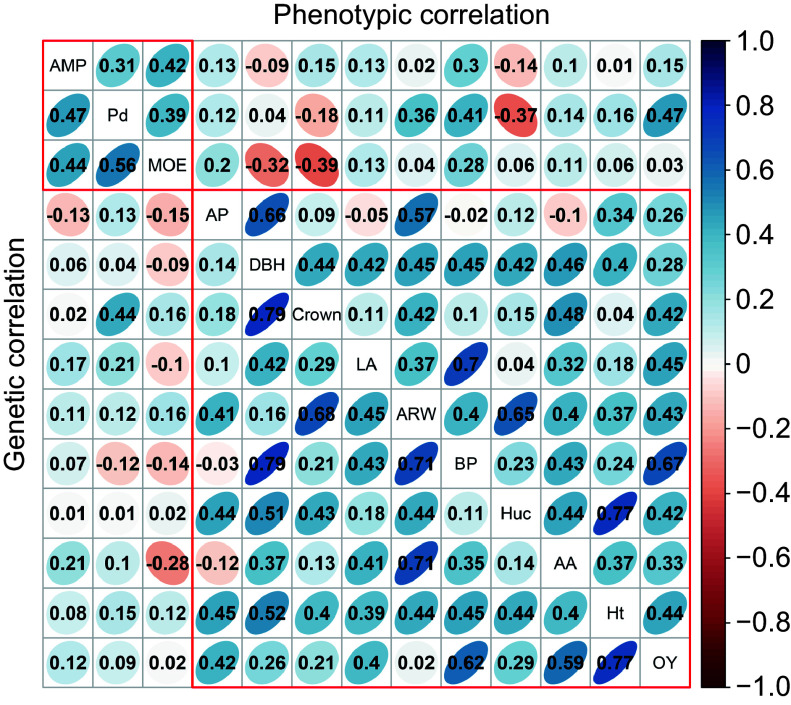
The analysis of phenotypic and genetic correlation within and among various traits. The upper triangular matrix displays phenotypic correlation, while the lower triangular matrix represents the genetic correlation results. AP, BP, AA, and LA represent Alpha-pinene, Beta-pinene, Abietic acid, and Levopimaric acid, respectively.

Further phenotypic and genetic correlation analyses were conducted among the 13 traits (Fig. 2). Positive correlations were observed within growth traits and wood properties, while some negative correlations were noted within resin traits. Notably, Beta-pinene showed the highest phenotypic and genetic correlations with OY (*r*_*p*_ = 0.62; *r*_*g*_ = 0.67), while Alpha-pinene exhibited negative phenotypic (*r*_*p*_ = −0.03) and genetic (*r*_*g*_ = −0.02) correlations with Beta-pinene and Abietic acid. Among wood properties and resin traits, the lowest phenotypic correlation was between MOE and abietic acid (*r*_*p*_ = −0.28). Among growth and resin traits, the highest phenotypic correlation was between DBH and Beta-pinene (*r*_*p*_ = 0.79), with a higher genetic correlation (*r*_*g*_ = 0.45). In addition, negative phenotypic (*r*_*p*_ = −0.32) and genetic (*r*_*g*_ = −0.09) correlations were observed with DBH and MOE.

### Estimation of breeding value of Slash pine

The BVs for growth traits are outlined in Supplemental Table S2. Family 8-126 showed the highest BV (0.719) for DBH, whereas family 11-26 had the lowest (BV = −0.821). Elite families for DBH, selected at a 20% rate, included 8-126, 8-131, 4-49, 7-258, 0-53, 2-296, and 0-1339. Similarly, elite families for Ht comprised 0-1027, 0-53, 8-126, 0-1339, 7-258, 10-105, and 11-6. For Huc, selected elite families were 3-1, 2-90, 0-636, 8-126, 7-258, 10-73, and 5-12. For ARW, the top 20% selected families were 2-296, 8-13, 11-6, 8-49, 3-1, 0-636, and 10-73. Supplemental Table S3 presents the BVs for wood properties. For MOE, selected elite families were 2-325, 0-464, 0-53, 0-373, 5-12, 4-9, and 11-26. Selected elite families for AMP included 2-325, 10-105, 8-126, 0-465, 0-1339, 4-49, and 0-1077. Supplemental Table S4 provides BVs for resin traits. The top 20% selected families for OY were 0-53, 7-77, 3-1, 0-1339, 8-131, 2-325, and 3-1. Alpha-pinene had the highest BV (1.314) in family 7-77 and the lowest BV (−1.226) in family 2-101. Selected families for Alpha-pinene were 7-77, 8-131, 7-258, 0-373, 0-636, 0-510, and 0-1027. Families selected for Beta-pinene matched those for Alpha-pinene.

### Multi-trait combined selection elite families of Slash pine

**Figure 3:**
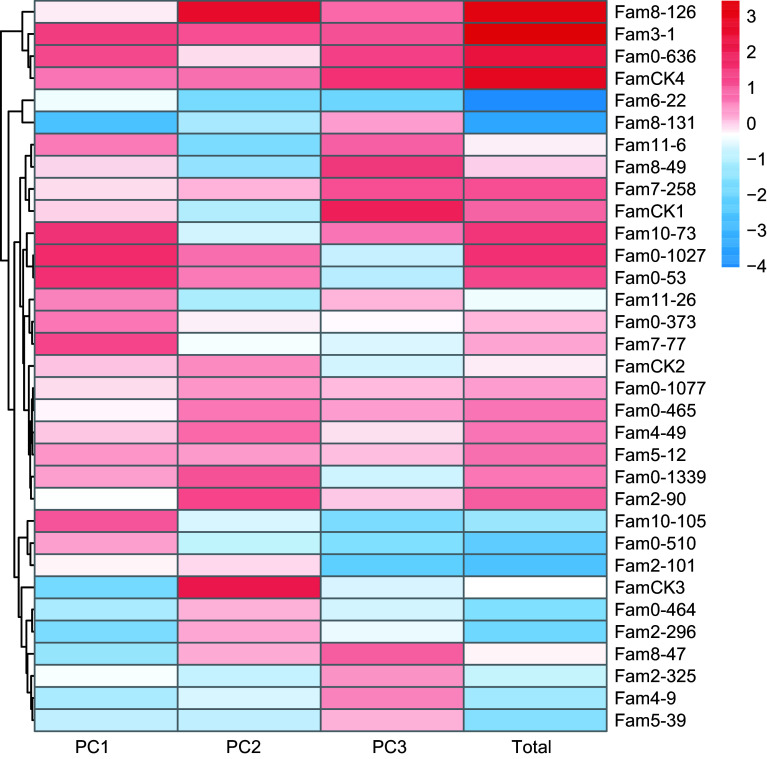
PC factor scores of growth traits in *P. elliottii* 33 half-sib families. The color of the squares transitions from blue to red, indicating increasing PC scores.

The results of PCA for growth traits are summarized in Table 3. Following the principle of achieving a cumulative contribution rate of over 85%, the study selected the top three PCs, which accounted for 91.5% and represented the main features of five growth traits. The eigenvalues for these three PCs were 1.689, 1.088, and 0.944, with contributions of 66.4%, 17.3%, and 7.7%, respectively. The weighted sum of individual PC scores, calculated based on the proportion of their eigenvalues to the total selected eigenvalues was utilized to determine the comprehensive scores for growth traits. The comprehensive evaluation model was defined as: *F*_*Total*_ = 0.454*F*_*1*_ + 0.292*F*_*2*_ + 0.254*F*_*3*_. Utilizing this model, comprehensive scores for each family were computed (Fig. 3), ranging from −4.032 to 3.431. The top seven families, identified as elite for growth traits based on a 20% selection rate, were 3-1, 8-126, 2-90, 0-636, 0-1027, 10-73, and 0-53.

**Table 3:** PCA of growth traits of *P. elliottii* half-sib families.

Summary	Traits	PCs		
		PC1	PC2	PC3
Feature vector	DBH	−0.457	0.654	0.000
	Ht	0.740	0.322	−0.300
	Huc	0.425	0.695	0.177
	Crown	−0.769	0.246	0.249
	ARW	0.400	−0.116	0.872
Eigenvalue		1.689	1.088	0.944
Contribution rate (%)		0.664	0.173	0.077
Cummulative contribution rate (%)		0.664	0.837	0.915

**Figure 4:**
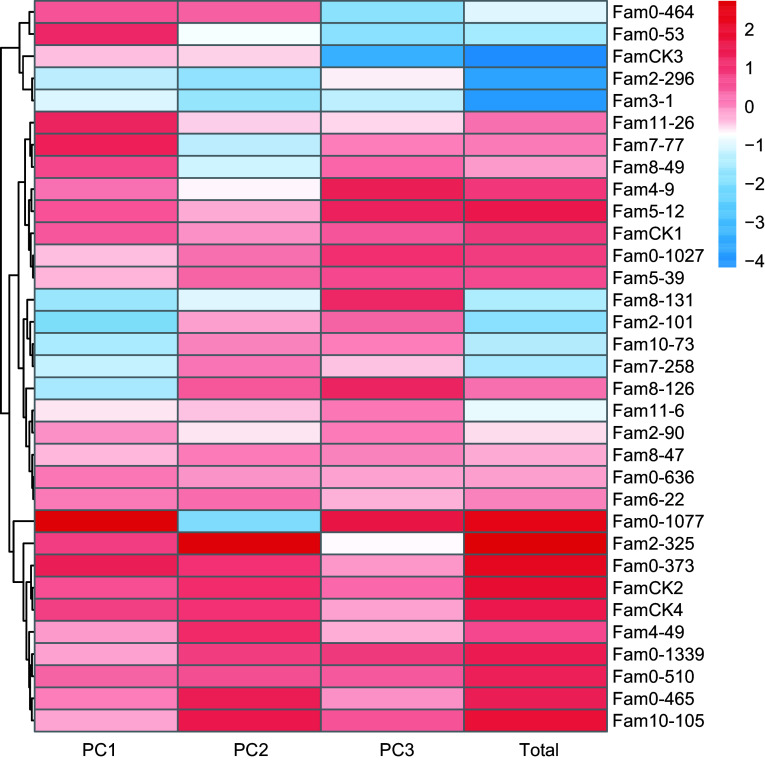
PC factor scores of wood quality traits in *P. elliottii* 33 half-sib families. The color of the squares transitions from blue to red, indicating increasing PC scores.

For wood properties, PCA results are presented in Table 4. The cumulative contribution rate of the top three principal components reached 100%, representing all features of the three wood properties. Eigenvalues for these PCs were 1.182, 0.984, and 0.833, contributing 39.4%, 32.8%, and 27.8%, respectively. The comprehensive evaluation model for wood properties was defined as: *F*_*Total*_= 0.394*F*_*1*_+ 0.328*F*_*2*_ + 0.278*F*_*3*_. Comprehensive scores ranged from −4.167 to 2.730 (Fig. 4), with elite families identified as 2-325, 0-1077, 0-373, 0-465, 10-105, 5-12, and 0-510.

**Table 4:** PCA of wood properties of *P. elliottii* half-sib families.

Summary	Traits	PCs		
		PC1	PC2	PC3
Feature vector	Pd	0.750	0.000	0.659
	MOE	0.673	0.464	−0.576
	AMP	−0.409	0.875	0.260
Eigenvalue		1.182	0.984	0.833
Contribution rate (%)		0.394	0.328	0.278
Cummulative contribution rate (%)		0.394	0.722	1.000

**Figure 5:**
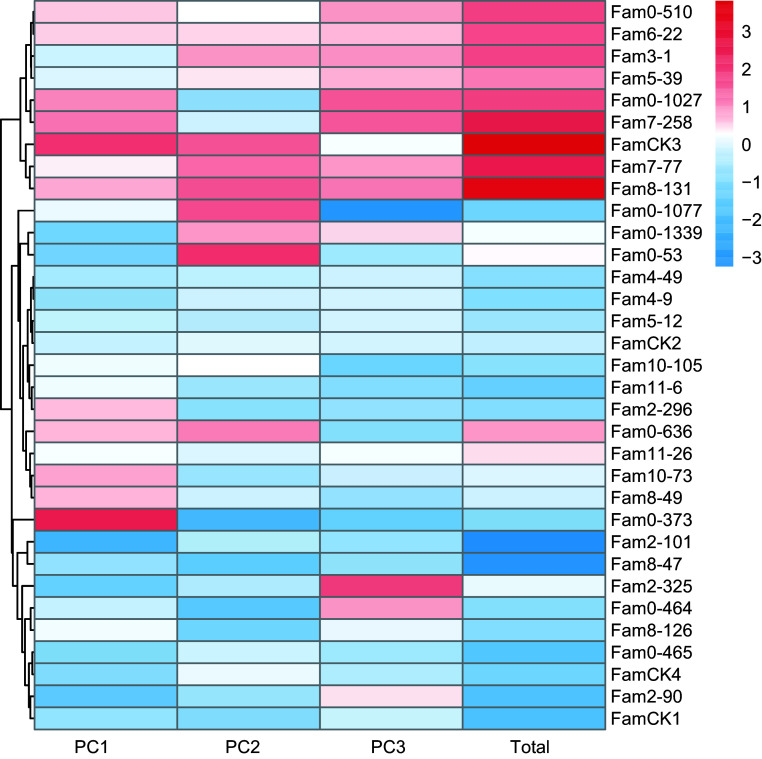
PC factor scores of resin traits in *P. elliottii* 33 half-sib families. The color of the squares transitions from blue to red, indicating increasing PC scores.

The PCA results for resin traits are presented in Table 5, where the cumulative contribution rate of the top three PCs reached 91.5%. Eigenvalues for these PCs were 2.290, 1.291, and 0.993, contributing 45.8%, 25.8%, and 19.9%, respectively. The comprehensive evaluation model for resin traits was defined as: *F*_*Total*_ = 0.501*F*_*1*_ + 0.282*F*_*2*_ + 0.217*F*_*3*_. Comprehensive score for each family ranged from −3.243 to 3.816 (Fig. 5), with elite families identified as 0-1077, 8-131, 7-258, 7-77, 0-1027, 0-510, and 3-1, based on a 20% selection rate.

**Table 5:** PCA of resin traits of *P. elliottii* half-sib families.

Summary	Traits	PCs		
		PC1	PC2	PC3
Feature vector	OY	−0.334	0.717	0.494
	Alpha_pinene	0.934	0.330	0.000
	Beta_pinene	0.933	0.331	0.000
	Abietic_acid	−0.660	0.572	0.000
	Levopimaric_acid	0.000	−0.481	0.856
Eigenvalue		2.290	1.291	0.993
Contribution rate (%)		0.458	0.258	0.199
Cummulative contribution (%)		0.458	0.716	0.915

## Discussion

### Enormous genetic improvement potential in breeding populations of Slash pine

Genetic variations are pivotal, directly influencing genetic gains and guiding breeding strategies in forests^[12]^. The study revealed significant differences among different family lines in traits. Growth traits were notably more influenced by environmental factors, whereas timber traits were primarily shaped by familial effects and resin traits were impacted by both family and environment in comparable proportions. These findings align with previous research on Slash pine^[18]^ and Japanese black pine^[52,53]^. Crown, AMP, and Beta-pinene display higher variability in this study when contrasted to Korean pine, whereas DBH and Ht show comparatively lower variations^[54]^.

Additionally, the heritability of growth traits in Slash pine surpassed previous findings^[18]^, while that of wood properties was slightly higher than those related to radiata pine^[55]^, suggesting a stronger genetic influence on wood properties compared to growth traits. Family heritability estimates for monoterpenes were akin to the study of Lei et al.^[56]^ but slightly lower than the result of Li et al.^[57]^, likely influenced by location-specific factors. Moreover, Ht, Pd, and Beta-pinene exhibit higher genetic gains at varying selection rates and intensities, slightly exceeding those reported in previous studies for growth and wood properties^[18,58]^, but demonstrating lower gains for resin traits^[12]^.

### Correlations analysis informs breeding strategy for Slash pine

In practical breeding, considering multiple traits simultaneously is essential for selecting improved genes, yet past studies indicate a trade-off between growth and wood quality^[59]^, underscoring the need for a comprehensive understanding of trait interrelationships to devise rational breeding strategies. This study reveals either non-significant or negative correlations between growth and wood properties, consistent with findings in other tree species like Norway spruce^[60]^ and poplar^[61]^, posing a significant challenge to genetic improvement in forestry^[62]^. Additionally, selecting trees at different growth stages may yield varying results^[63]^. Correlative research on Japanese black pine identified significant genetic correlations between growth, morphological, and OY, such as a correlation of 0.73 between DBH and OY^[53]^. Similarly, this study shows comparable results, with a correlation of 0.79 between DBH and Beta-pinene content, and 0.77 between Ht and resin yield, possibly due to vigorous growth promoting resin duct formation^[64,65]^. Furthermore, weak positive or negative correlation between wood properties and resin traits, uncommon in other tree species, were observed. Overall, breeders must meticulously consider trait correlations when selecting for improved genes, and the observed correlations in Slash pine offer valuable insights for effective breeding strategies.

### Substantial achievable genetic gains in elite families of Slash pine

For a considerable duration, combined selection of multiple traits has been central in both animal and plant breeding^[66,67]^, with models addressing the impact of LD between traits. PCA, a multivariate selection technique, facilitates the exploration of relationships between explanatory variables and correlated traits^[68]^. In this study, a combined approach using single-trait and PCA-based selection identified elite families for growth, wood properties, and resin traits. Achievable average genetic gains for the three trait categories were 7.4%, 7.2%, and 8.8%, respectively, consistent with a prior study on Slash pine^[18]^. Genetic gains for growth traits exceeded the 10% for Scots pine, while those for wood properties were comparable to its WD related traits^[69]^. However, genetic gains for resin traits were lower than those reported for Slash pine^[45]^. Quantitative traits are subject to varying degrees of gene-environment interactions, with diverse outcomes influenced by factors such as experimental design, the number of tested families, and different growth periods.

## Conclusions

This study conducted a comprehensive assessment of genetic variation among 33 half-sib families of Slash pine, aiming to identify elite families suitable for industrial use through a multi-trait combined selection approach. Significant differences were noted among families across 13 traits, with growth traits primarily influenced by block × family interaction, wood properties mainly affected by family effects, and resin traits showing variation attributed to both family effects and block × family interaction. Strong genetic control was evident for several traits, notably Beta-pinene, which exhibited the highest variations, and genetic gains, indicating significant breeding potential. Negative correlations were observed between growth and wood properties, while positive correlations were found between growth and resin traits. The multi-trait combined selection successfully identified elite families for growth, wood properties, and resin traits. This study provides important references for the long-term breeding strategies of Slash pine, offering rich genetic resources for genomic breeding and molecular breeding.

## SUPPLEMENTARY DATA

Supplementary data to this article can be found online.
