# Supplementary material for: Genetic selection for growth, wood quality and resin traits of potential Slash pine for multiple industrial uses
Source: For Res (Fayettev). 2024 Jun 20;4:e023. doi: 10.48130/forres-0024-0020 (PMC11524238; doi:10.48130/forres-0024-0020)
Supplement: Supplementary file 1 — Supplementary data to this article can be found online. [file forres-0024-0020-S1.zip › 10.48130_forres-0024-0020-Suppl-TableS2.pdf]

Supplemental Table S2 Estimated breeding value for growth traits

Families	DBH	Ht	Huc	Crown	ARW
0-1027	0.24	0.29	0.03	-0.11	0.04
0-1077	0.27	0.07	-0.08	0.02	0.03
0-1339	0.33	0.18	0.02	0.00	-0.05
0-373	-0.26	0.00	0.10	-0.05	-0.02
0-464	0.28	-0.03	-0.15	0.05	-0.12
0-465	0.32	-0.10	0.11	-0.02	0.00
0-510	-0.44	0.05	-0.08	-0.04	-0.18
0-53	0.44	0.26	-0.03	-0.15	0.03
0-636	-0.45	-0.03	0.27	-0.04	0.16
10-105	-0.57	0.12	0.03	-0.06	-0.16
10-73	-0.55	0.08	0.10	-0.06	0.15
11-26	-0.82	0.02	0.02	0.03	0.01
11-6	-0.56	0.10	-0.27	0.01	0.19
2-101	-0.07	-0.08	0.05	-0.07	-0.30
2-296	0.35	-0.17	-0.09	0.07	-0.15
2-325	-0.11	-0.13	-0.09	-0.01	0.03
2-90	0.01	-0.07	0.32	0.05	-0.10
3-1	0.08	0.09	0.33	-0.06	0.16
4-49	0.53	0.01	0.03	-0.05	0.00
4-9	0.04	-0.20	-0.11	0.04	0.00
5-12	-0.17	0.05	0.12	0.02	0.00
5-39	0.15	-0.17	-0.21	-0.01	0.00
6-22	-0.43	0.09	-0.43	0.01	-0.19
7-258	0.25	-0.08	0.01	-0.01	0.13
7-77	-0.24	0.04	0.09	-0.12	-0.01
8-126	0.72	0.19	0.23	0.12	0.07
8-131	0.71	-0.28	-0.56	0.07	-0.01
8-47	0.00	-0.22	0.09	0.11	-0.02
8-49	-0.27	-0.17	-0.12	-0.02	0.17
CK1	-0.26	-0.09	-0.10	0.04	0.24
CK2	-0.05	0.14	0.01	0.05	-0.08
CK3	0.49	-0.06	0.22	0.06	-0.24
CK4	0.08	0.08	0.15	0.02	0.21
