# Supplementary material for: Genetic selection for growth, wood quality and resin traits of potential Slash pine for multiple industrial uses
Source: For Res (Fayettev). 2024 Jun 20;4:e023. doi: 10.48130/forres-0024-0020 (PMC11524238; doi:10.48130/forres-0024-0020)
Supplement: Supplementary file 1 — Supplementary data to this article can be found online. [file forres-0024-0020-S1.zip › 10.48130_forres-0024-0020-Suppl-TableS3.pdf]

Supplemental Table S3 Estimated breeding value for wood properties

Families	Pd	MOE	AMP
0-1027	0.24	0.29	0.03
0-1077	0.27	0.07	-0.08
0-1339	0.33	0.18	0.02
0-373	-0.26	0.00	0.10
0-464	0.28	-0.03	-0.15
0-465	0.32	-0.10	0.11
0-510	-0.44	0.05	-0.08
0-53	0.44	0.26	-0.03
0-636	-0.45	-0.03	0.27
10-105	-0.57	0.12	0.03
10-73	-0.55	0.08	0.10
11-26	-0.82	0.02	0.02
11-6	-0.56	0.10	-0.27
2-101	-0.07	-0.08	0.05
2-296	0.35	-0.17	-0.09
2-325	-0.11	-0.13	-0.09
2-90	0.01	-0.07	0.32
3-1	0.08	0.09	0.33
4-49	0.53	0.01	0.03
4-9	0.04	-0.20	-0.11
5-12	-0.17	0.05	0.12
5-39	0.15	-0.17	-0.21
6-22	-0.43	0.09	-0.43
7-258	0.25	-0.08	0.01
7-77	-0.24	0.04	0.09
8-126	0.72	0.19	0.23
8-131	0.71	-0.28	-0.56
8-47	0.00	-0.22	0.09
8-49	-0.27	-0.17	-0.12
CK1	-0.26	-0.09	-0.10
CK2	-0.05	0.14	0.01
CK3	0.49	-0.06	0.22
CK4	0.08	0.08	0.15
