# Supplementary material for: Genetic selection for growth, wood quality and resin traits of potential Slash pine for multiple industrial uses
Source: For Res (Fayettev). 2024 Jun 20;4:e023. doi: 10.48130/forres-0024-0020 (PMC11524238; doi:10.48130/forres-0024-0020)
Supplement: Supplementary file 1 — Supplementary data to this article can be found online. [file forres-0024-0020-S1.zip › 10.48130_forres-0024-0020-Suppl-TableS4.pdf]

Supplemental Table S4 Estimated breeding value for resin traits

Families	OY	Alpha_pinene	Beta_pinene	Abietic_acid	Levopimaric_acid
0-1027	0.00	0.45	0.54	-0.06	0.08
0-1077	-0.01	0.23	0.27	0.04	-0.16
0-1339	0.03	-0.41	-0.49	0.06	0.00
0-373	-0.06	0.69	0.82	-0.18	-0.03
0-464	-0.02	-0.32	-0.38	-0.02	0.08
0-465	0.00	-0.58	-0.69	0.02	-0.02
0-510	0.00	0.46	0.54	0.01	0.04
0-53	0.05	-0.30	-0.36	0.07	-0.08
0-636	0.00	0.55	0.65	0.02	-0.06
10-105	-0.03	0.17	0.20	0.04	-0.04
10-73	-0.03	0.35	0.42	-0.02	0.02
11-26	0.01	0.08	0.10	-0.02	0.00
11-6	-0.04	-0.01	-0.02	0.00	-0.01
2-101	0.00	-1.23	-1.46	0.05	-0.02
2-296	-0.05	0.25	0.30	0.00	0.00
2-325	0.03	-0.66	-0.79	0.04	0.09
2-90	0.01	-0.91	-1.08	0.03	0.03
3-1	0.05	0.07	0.08	0.00	0.01
4-49	0.00	-0.33	-0.39	0.00	0.00
4-9	0.01	-0.47	-0.56	0.00	-0.01
5-12	0.00	-0.22	-0.26	-0.02	0.00
5-39	0.02	0.12	0.14	0.01	0.02
6-22	0.02	0.39	0.47	-0.01	0.01
7-258	0.00	0.72	0.85	-0.02	0.07
7-77	0.05	0.40	0.47	0.00	-0.01
8-126	-0.02	-0.12	-0.15	-0.04	0.03
8-131	0.03	0.84	1.01	0.05	0.02
8-47	-0.04	-0.65	-0.77	0.00	0.01
8-49	-0.04	0.40	0.48	0.01	-0.01
CK1	0.00	-0.65	-0.77	-0.04	0.00
CK2	0.00	-0.09	-0.11	0.01	0.00
CK3	0.02	1.31	1.56	-0.04	-0.03
CK4	0.01	-0.53	-0.63	0.02	-0.03
